# State Resident Handedness, Ideology, and Political Party Preference: U.S. Presidential Election Outcomes Over the Past 60 Years

**DOI:** 10.1177/00332941241227521

**Published:** 2024-01-12

**Authors:** Stewart J. H. McCann

**Affiliations:** Department of Psychology, 55964 Cape Breton University, Sydney, NS, Canada

**Keywords:** Handedness, liberal, conservative, Republican, Democrat, presidential elections

## Abstract

Pearson correlation, partial correlation, and multiple regression strategies determined the degree to which estimates of the level of left-handedness in each of the 48 contiguous American states related to citizen political ideology and to Democratic-Republican presidential popular vote over the past 60 years. Higher state levels of left-handedness were associated significantly with liberal ideology in each of the presidential election years from 1964 to 2016. Comparable ideology data were not available for 2020. Higher state levels of left-handedness also were associated with a greater degree of Democratic candidate popular vote support in each of the presidential election years from 1964 to 2020 except for 1976. The mean size of these 28 significant Pearson correlations involving the two political criteria was .62 (*SD* = .12) with a range of .38–.80, indicating handedness alone could account for a mean of 40.1% (*SD* = 14.9) of the variance in the two political preference variables. Corresponding multiple regressions showed that when state-level Big Five personality, White population percent, urbanization, and income variables were given the opportunity to enter the equations, handedness still emerged with a significant regression coefficient in 26 of the 28 equations. The two exceptions occurred for 1968 with either political preference criterion. It is speculated that such relations are grounded in hypothesized but poorly understood genetic links between handedness, personality, and political beliefs and attitudes, and, that a foundational *genetic predisposition* to left-handedness in a population may have much greater impact on correlates than *overt* levels of left-handedness.

## Introduction

It seems intuitively apparent that right- and left-handedness should not be associated with being “right” or “left” on the political spectrum. Yet, that is what was found by McCann (2018a). In that research conducted from the geographical psychology perspective (e.g., Rentfrow et al., 2008; Rentfrow & Jokela, 2016) with the 48 contiguous American states as analytical units, the Pearson correlation was an extremely high −.80 between an index of state resident left-handedness provided by McManus (2009) and a state composite measure of Conservative-Republican ideological and party preference based on the period from 1996 to 2012. This robust association also was maintained when state socioeconomic status (SES), White population percent, and urban population percent were statistically controlled in multiple regression equations.

Of course, the exceptional magnitude of the relation between handedness and political preference reported by McCann (2018a) should be interpreted within the context of aggregation. Analyses of variables based on aggregated units such as states generally produce larger correlations and regression coefficients than analyses based on variables with individuals as the analytical units (e.g., Erikson et al., 1993; Ostroff, 1993). This occurs because the aggregation process tends to cancel out the degree of measurement error for each aggregated unit and increases reliability.

Surprisingly, McCann (2018a) also found that each of the Big Five personality dimensions assessed at the state level (Rentfrow et al., 2008) correlated significantly with both the Conservative-Republican preference variable *and* with handedness, but in opposite directions. Greater Conservative-Republican preference was associated with lower neuroticism and openness to experience, and was associated with higher conscientiousness, extraversion, and agreeableness. In contrast, a tendency for a state to have left-handed residents was associated with *higher* neuroticism and openness to experience, and was associated with *lower* conscientiousness, extraversion, and agreeableness. Furthermore, when handedness was controlled in a multiple regression equation, all relations between Conservative-Republican preference and the Big Five were eliminated. To McCann (2018a), the fact that all relations between the Big Five and Conservative Republican preference were erased when handedness was controlled suggested the presence of underlying but largely unknown genetic links between personality, political preference, and handedness.

Although their nature is not well understood, there is general agreement that handedness has genetic determinants (e.g., Cuellar-Partida et al., 2021; Schmitz et al., 2018; Wiberg et al., 2019). There also is longstanding evidence that the Big Five personality variables have strong genetic underpinnings (e.g., Jang et al., 1996; Loehlin et al., 1998; Vukasovic & Bratko, 2015). As well, many researchers have proposed that political orientation depends to some extent on genetic foundations (e.g., Dawes & Weinschenk, 2020; Hufer et al., 2020; Schwabe et al., 2016).

The largest study of the genetic basis of handedness thus far was reported by Cuellar-Parida et al. (2021) based on a genome-wide meta-analysis of 1,266,671 persons conducted by 98 authors. They located 41 loci associated with left-handedness, each increasing the chances of left-handedness by a small increment. Cuellar-Parida et al. (2021) concluded that “the present findings firmly support the hypothesis that handedness, like many other behavioural and neurological traits, is influenced by many variants of small effect and multiple biological pathways” (pp. 63–64).

Researchers have consistently found that the Big Five personality variables are moderately to highly heritable. Vukasovic and Bratko (2015) conducted a meta-analysis and found that the mean effect size for genetic variance in descending order was 41% for openness to experience, 37% for neuroticism, 36% for extraversion, 35% for agreeableness, and 31% for conscientiousness. Earlier, Jang et al. (1996) gauged the heritability of the Big Five among 123 pairs of identical twins and 127 pairs of fraternal twins and found that the corresponding heritability effect sizes were 61% for openness to experience, 41% for neuroticism, 53% for extraversion, 41% for agreeableness, and 44% for conscientiousness. Similarly, Loehlin et al. (1998) tested 490 pairs of identical twins and 317 pairs of fraternal twins and found that the corresponding heritability effect sizes were 56% for openness to experience, 58% for neuroticism, 57% for extraversion, 51% for agreeableness, and 52% for conscientiousness.

Dawes and Weinschenk (2020) reviewed the literature on the genetics of political orientation and concluded that it has quite consistently been found to be about 40% heritable. However, they suggest that the twin studies, such as the one by Alford et al. (2005), on which most heritability contributions are based may underestimate the influence of genetics on ideology. They also point out that no study has provided clear evidence of any specific genetic marker associated with ideology.

However, empirical research findings to support McCann’s (2018a) speculation regarding underlying genetic links between Big Five personality, political orientation, and handedness is extremely limited. In fact, little or no research has been conducted that can offer support or disconfirmation for such speculated underlying genetics common to personality and political preference, to personality and handedness, to political preference and handedness, and to personality, political preference, and handedness. Researchers have found that there are some genetic features that are common to both Big Five personality traits and political orientation (e.g., Eaves et al., 1989; Kandler et al., 2015; Verhulst et al., 2012). As well, Cuellar-Partida et al. (2021) reported in their Supplementary Table 4 that neuroticism showed relations to several single nucleotide polymorphisms (SNPs) that they found were also related to handedness. However, there does not appear to have been any research conducted regarding the commonly held genetic features that might exist for political preference and handedness, or for personality, political preference, and handedness.

In offering speculations regarding potential explanations for the relations between handedness, the Big Five, and political orientation, McCann (2018a) emphasized that it is not warranted to assume from the state-level results that handedness is likely to predict political preference when individuals are the analytic units. Everyday experience makes it quite clear that the demonstrated state-level relations “do not exist simply because right-handers tend to be less open, less neurotic, and more conservative” (p. 305). If that were true, “then such handedness connections would be noticeably large, easily observable in social interactions, and certainly evident in individual-level studies” (p. 305).

Instead, other factors were speculated to be involved in forming the links between handedness, personality, and political preference. One intriguing possibility suggested by McCann (2018a) is that “latent or hidden relations may exist between handedness, political orientation, and personality that depend upon both the direct and more tangential genetics of handedness” (p. 308). What is *speculated* is that apparent left-handedness may be only one manifestation of a genetic network underlying relations between handedness, personality, and political persuasion. Other influences of a genetic or nongenetic nature might inhibit observed left-handedness but still leave the foundational genetic network responsible for the relations intact. Consequently, the population of *genetically predisposed* “left-handers” in a state may be grossly underestimated. McCann (2018a) expressed this possibility as an “iceberg effect” in which “what is hidden might have a much greater effect on political orientation and personality than what would be expected from relative differences in observed levels of left-handedness in different geographical areas” (p. 308).

Although the present research focuses on the Big Five personality variables, it is possible that other dispositional factors also are implicated in relations between handedness and political preference. Two candidates likely at the fore in this respect are personality dimensions based on right-wing authoritarianism (RWA; Altemeyer, 1988) and social dominance orientation (SDO; Pratto et al., 1994). Both higher RWA and higher SDO are associated with conservative political preferences and attitudes toward a variety of attitude objects such as racial differences, outgroups, and social justice reforms (e.g., Cuevas & Dawson, 2020; Ho et al., 2012). Both RWA and SDO also have been found to have genetic foundations that are shared to some degree with attitudes embedded in conservative ideology (e.g., Kleppesto et al., 2019; Lewis & Bates, 2014).

Several studies have reported that RWA or authoritarian submission is associated with consistent rather than inconsistent hand preference (e.g., Chan, 2018; Christman, 2014; Grillo et al., 2018). Furthermore, Lyle and Grillo (2014, 2020) not only found that consistent handedness was associated with authoritarian submission, but also that consistent handedness was associated with greater expressed prejudice toward out-groups such as liberals, homosexuals, atheists, and immigrants. As well, Lyle and Grillo (2014) found that consistent handedness was associated with preference for the conservative Republican party, and Prichard and Christman (2020) found that consistent handedness was associated with stronger support for Donald Trump during the 2016 Republican presidential primary.

These results become more important in the current context when it is understood that consistent versus inconsistent handedness generally reduces to consistent right-handers versus inconsistent right-handers because of the overwhelming preponderance of right-handers in the samples employed and in the populations from which they are drawn (Hardie & Wright, 2014). It seems conceivable that many “right-handers” who are less committed to the sole use of their dominant right hand might be speculated to be displaying evidence of what McCann (2018a) refers to as “latent left-handedness.” Within this interpretive context, although not intended to do so, the preceding research (i.e., Chan, 2018; Christman, 2014; Grillo et al., 2018; Lyle & Grillo, 2014, 2020; Prichard & Christman, 2020) may have provided empirical data suggesting that right-handed persons are more likely to exhibit higher authoritarian tendencies, more prejudice toward out-groups, and greater support for the conservative Republican side of the political spectrum. No research seems to exist that has assessed both SDO and handedness, but perhaps similar relations might be found regarding common underlying genetics.

Whatever the underlying processes and dynamics, it is important to consider that the McCann (2018a) research is the only *published* study reporting empirical relations between state resident handedness, the Big Five, and political leaning. However, it was later learned by McCann through the peer review process and acknowledged in the article that somewhat similar relations had been explored in an unpublished work. Dr Chris McManus of University College London had delivered a presentation titled “Genes, Geography, and Handedness” at the Tarragon Laterality Conference in Tarragona, Spain, on February 11, 2013.

The present study and the earlier one by McCann (2018a) are grounded in the geographical psychology approach (e.g., Rentfrow, 2010, 2014; Rentfrow et al., 2008; Rentfrow & Jokela, 2016). A fundamental tenet of the perspective is that the aggregate position on an individual difference variable in a locale taps the central tendency of that locale’s residents, and that it is associated with the pervasiveness in that locale of the behavioral and psychological tendencies associated with that individual difference variable. Geographical psychology derives much in the way of its research questions and directions from psychological theory and empirical results pertaining to individuals, but it also is thought to offer the possibility of providing important and supplementary knowledge to conventional individual difference psychology in return (e.g., Chen, 2020; Rentfrow, 2010). To Rentfrow and Jokela (2019), aggregate-level studies are beneficial “because they can indicate the degree to which psychological processes generalize across multiple levels of analysis and cultures” (p. 778) and their “results have the potential to reveal whether psychological characteristics contribute to important macro-level outcomes” (p. 778).

Rentfrow (2010) also suggests that geographical psychology research based on aggregates as the analytic units can lead us to a more nuanced understanding of various psychological constructs than might be found solely with conventional psychological research that uses individuals as the analytic units. For example, research using individuals as the analytic units conducted in one geographical locale might produce different results than the same study conducted in a different geographical locale because differing social norms are prevalent in each of the two places. Therefore, findings from individual-level psychological research may at least sometimes be less universal and more tainted by geographical locale than usually thought. Rentfrow and Gosling (2021) state that “if we are to develop a thorough understanding of personality processes, we must consider the broader environment in which those processes unfold” (p. 824). Therefore, the geographical psychology perspective promotes a more comprehensive investigation of the societal role of personality based on geographical units such as states, and the relation of that role to personality theory and empirical evidence as conventionally researched with individuals as the units of analysis.

The present research emphasizes the *genetic* underpinning of handedness, personality, and political preference, although it is also granted that these constructs may be influenced to some degree by environmental factors. The general emphasis on *genetic* foundations is compatible with two prevailing views regarding the development of personality differences that draw on arguments initially pertaining to cultural impacts on personality. Rentfrow (2010) points out, that some (e.g., McCrae & Costa, 1996) see personality traits as *genetically* driven tendencies, and therefore any differences at an aggregate or cultural level of analysis also are assumed to be based on *genetic* differences between the aggregate or cultural units (see Hofstede & McCrae, 2004, pp. 74–78). In contrast, others (e.g., Saucier & Goldberg, 1996) see personality traits as based on both *genetics* and environmental factors, and therefore any differences at an aggregate or cultural level of analysis also are assumed to be based on both *genetic* and environmental differences between the aggregate or cultural units (see Hofstede & McCrae, 2004, pp. 74–78). Similar dynamics probably can be applied to the development of political preference. It is the view of the current author that personality is quite stable and rooted in genetics, and that environmental factors are much more likely to influence the expression of a personality trait rather than alter the nature of the trait itself.

### The Present Study

The present research was initiated to determine the degree of replicability of the McCann (2018a) results pattern when the U.S. presidential election years from 1964 to 2020 are analyzed separately. The period from 1964 to 2020 was chosen for the present study because the passing of the Civil Rights Act of 1964 signaled the beginning of a major political party realignment that largely converted the southern states from Democratic to Republican preference (e.g., Beck, 1977; Black, 2004), a shift that has lasted until 2020 and beyond. This relatively stable period of state political orientation includes the years from 1996 to 2012 on which the McCann (2018a) composite measure of Conservative-Republican preference was based. Therefore, if the results pattern found by McCann (2018a) could be replicated for individual presidential elections, it could be assumed that it would be most likely to occur for the elections from 1964 to 2020 rather than for earlier elections.

There also has been growing political polarization in the USA since 1964. During this time, “conservative” policies have been largely supported by the Republicans and “liberal” policies by the Democrats (e.g., Jost, 2006). However, these ideology-party alignments became increasingly pronounced from 1964 to 2020 (e.g., Abramowitz & Saunders, 1998; Wright & Birkhead, 2012). This strengthening alignment has occurred largely because Democratic-Republican partisanship which is more fluid has been continuously fine tuned to correspond to Conservative-Liberal ideology which is relatively more stable than party preference (Wright & Birkhead, 2012).

This conclusion also is consistent with the results of Fazekas and Littvay (2015) showing that there is a genetic correlation between ideology and party preference, the results of Alford et al. (2005) showing that genetics has a stronger role regarding ideology than party preference, and the results of Bell and Kandler (2015) showing that genetic and environmental factors influence political ideology which then affects party preference. In addition, it seems apparent that the rise of social media has enhanced the capacity to sort politically charged issues and positions so that they are aligned more easily with the *proper* party identification. Therefore, if the McCann (2018a) results pattern is to be replicated in individual presidential election years, then it would seem most likely to happen when there is marked separation between Democratic and Republican Party preference, as in the 1964 to 2020 period.

The results produced by McCann (2018a) relied on only one composite assessment of Conservative-Republican preference. It was based jointly on resident self-identification as liberal or conservative in each of the 48 contiguous states from national *CBS*/NYT phone polls conducted from 1999–2003 (Wright, 2012), “conservative advantage” in each state defined as the mean percentage of conservatives minus the mean percentage of liberals taken from a Gallup national phone survey conducted throughout 2012 (Newport, 2013), and the mean of the percentages of each state population voting for the Republican presidential candidate in the elections of 1996, 2000, 2004, 2008, and 2012 (Leip, 2017). Whether the overall relations between handedness, personality, and political orientation found by McCann (2018a) also pertain to each of the presidential elections in the period from 1996 to 2012 cannot be ascertained from that research report.

The present study focuses separately on state ideology and state political party preference. The research design allowed for the multivariate prediction of each of these two political outcome variables by handedness, the Big Five personality variables, income, race, and urbanization for each presidential election year from 1964 to 2020. The prediction of political party preference by ideology when handedness, personality, income, race, and urbanization were considered as covariates also was addressed for each of the 15 election years.

In the present research, it was generally expected that handedness would be a predictor with ideology as the criterion because it predicted the composite ideology-partisanship criterion in the McCann (2018a) study. Such a relation also might occur if, as suggested by earlier research, political orientation has some genetic basis (e.g., Dawes & Weinschenk, 2020; Hufer et al., 2020; Schwabe et al., 2016), handedness is genetically determined (e.g., Cuellar-Partida et al., 2021; Schmitz et al., 2018; Wiberg et al., 2019), and the speculated genetic links between political orientation and handedness by McCann (2018a) are at least somewhat veridical.

Furthermore, a relation between handedness and political ideology might exist at least in part if handedness relates to personality (McCann, 2018a) and Big Five personality relates to Conservative-Liberal ideology (e.g., Carney et al., 2008; Gerber et al., 2012; McCann, 2014a). It seems fairly evident that political ideology defined as “an interrelated set of moral and political attitudes that possesses cognitive, affective, and motivational components” (Jost, 2006, p. 653) should be related to important personality factors such as those in the Big Five model. As well, there might be genetic links between handedness and personality that are implicated in relations between handedness and political ideology. McCann (2018a) cited research suggesting that left-handers have superior interhemispheric interaction (e.g., Cherbuin & Brinkman, 2006), and that poorer interhemispheric interaction based on presumably genetically influenced corpus callosum differences (e.g., Luders et al., 2010; Propper et al., 2012) is associated with conservative tendencies such as cognitive inflexibility and lack of tolerance (e.g., Cherbuin & Brinkman, 2006).

For similar reasons, it also was expected that Democratic-Republican preference would be predicted by handedness in the present study. Of course, handedness had predicted the composite ideology-partisanship criterion in the McCann (2018a) research. Earlier research also suggests that Democratic-Republican preference has a genetic basis (e.g., Alford et al., 2005; Bell & Kandler, 2015; Fazekas & Littvay, 2015) and that Big Five personality relates to Democratic-Republican preference (e.g., Barbaranelli et al., 2007; McCann, 2014b; Mondak & Halperin, 2008). Dynamics parallel to those postulated for the relation between handedness and ideological orientation are likely to apply to Democratic-Republican preference.

The analysis also determined whether political ideology still is a potent predictor of political party preference when handedness, the Big Five, income, race, and urbanization are considered as statistical controls. These covariates were expected to be predictors of political ideology. Therefore, the remaining capacity of political ideology as a predictor of political party preference with these factors evaluated as controls also was a question to be answered.

Big Five personality, income, race, and urbanization were considered in the current research as covariates in the evaluations of handedness as a predictor of ideological orientation and political party preference, but they also were of interest as predictors. There is state-level evidence that the Big Five are related to political ideology (e.g., McCann, 2014a), party preference (e.g., McCann, 2014b), and handedness (e.g., McCann, 2018a). Direct and indirect evidence also shows that income is related to political ideology and party preference (e.g., McCann, 2014b, 2018b; Muro & Whiton, 2019), and to handedness (e.g., Goodman, 2014; McCann, 2018). As well, race has been related to political ideology (e.g., Saad, 2022) and Democratic-Republican preference (e.g., McCann, 2018a), and urbanization has been related to political ideology (e.g., Kaufman, 2021; McCann, 2018c) and Democratic-Republican preference (e.g., McCann, 2018a). However, neither White nor urban population percent was related to handedness in the McCann (2018a) state-level study. Others have reported individual-level relations of handedness to race (e.g., Lansky et al., 1988), but no evidence could be located that showed a relation between handedness and urbanization. The Big Five, income, and race were considered as statistical controls in the present study because of their previously demonstrated relations to handedness *and* to the two political outcome variables. Urbanization also was included because of its potential values as a covariate and because it might be important as a variable of interest as an independent predictor in the present context.

The present research was conducted to determine whether the relations uncovered by McCann (2018a) could be generalized separately to each of the presidential election years from 1964 to 2000. It was not specifically designed to substantiate the accuracy of the speculative interpretations made by McCann (2018a), to elaborate those postulations, or to furnish novel suppositions. Therefore, underlying explanations for any associations found between handedness, Big Five personality, and political orientation in the present study understandably are bound to draw largely from the earlier interpretive speculations as stated by McCann (2018a).

## Method

### Measures

#### Democratic-Republican Percent of the Popular Vote

The percent of the popular vote won in each of the 48 contiguous states by the national Democratic and Republican presidential candidates in each of the elections from 1964 to 2020 was provided by Leip (2022). The key criterion variable in each of the planned analyses was developed by subtracting the Republican percent from the Democratic percent in each state for each year from 1964 to 2020.

#### Ideology

The state citizen ideology scores for each of the presidential election years from 1964 to 2016 were taken from Fording (2018). Scores for the years prior to 1996 were produced by Berry et al. (1998) and the work by Fording (2018) essentially used the same procedure to create the revised 1960-2016 citizen ideology series. The original series by Berry et al. (1998) measured citizen ideology by identifying “the ideological position of each member of Congress in each year using interest group ratings” (p. 330) provided by a rating organization, then estimating “citizen ideology in each district of a state using the ideology score for the district’s incumbent, the estimated ideology score for a challenger (or hypothetical challenger) to the incumbent, and election results that presumably reflect divisions in the electorate” (pp. 330–331). Finally, the district citizen ideology scores were “used to compute an unweighted average for the state as a whole” (p. 331). Incumbent ideology is determined by a rating organization, but challenger ideology is estimated as “equal to the average ideology of all incumbents in the state from the same party” (p. 331). Higher citizen ideology scores are associated with greater liberalism. Berry et al. (1998) reported information indicating that the state citizen ideology scores have high reliability, high validity of their explicit assumptions underlying the development of the scores, high convergent validity, and high construct validity.

#### Handedness

The percent of left-handers in each of the 48 contiguous states was taken from McManus (2009, p. 48). The steps in the development of these handedness values began with a 1986 *National Geographic* issue which contained a mail-in odor survey in which readers also were asked to indicate their throwing and writing hand (Gilbert & Wysocki, 1987). In an exploration of age and hand preference, Gilbert and Wysocki (1992) used data from 1,177,507 U.S. respondents 10–86 years of age who had indicated both their writing and throwing hand. McManus (2009) then selected from these respondents, all 412,923 left-handed White Americans born in 1950 or later who lived in the 48 contiguous states and the District of Columbia. Using respondent zip codes, McManus was able to compute the percent left-handed in each location.

#### Big Five Personality

Based on responses to the 44-item Big Five Inventory (John & Srivastava, 1999) of 619,397 respondents in an internet survey conducted between December of 1999 and January of 2005, Rentfrow et al. (2008) computed state z scores for neuroticism, extraversion, agreeableness, conscientiousness, and openness to experience. They concluded that the sample generally was representative of the national population, that the survey drew participants from each state in direct proportion to the 2000 census figures regarding population and racial composition, but that the sample was somewhat less representative regarding social class and was much younger than the general population. They also concluded that “the state-level factor structure was virtually identical to the factor structure commonly found at the individual level” (p. 349), and that the Big Five assessment was reliable with mean Cronbach alphas of .89 at the state level and .81 at the individual level. The present study used the original z scores based on the 50 states and Washington, DC.

#### Income

State economic condition estimates were represented by per capita personal income for the presidential election years from 1964 to 2020 taken from BEA (2022).

#### White Population Percent

The White population percent for each state was obtained for 1960 from U.S. Department of Commerce (1961), for 1970, 1980, and 1990 from Hobbs and Stoops (2002), for 2000 from U.S. Census Bureau (2002), for 2010 from KFF (2022), and for 2020 from U.S. Census Bureau (2021). Prorated White population percent estimates were created for 1964, 1968, 1972, 1976, 1984, 1988, 1992, 1996, 2004, 2008, 2012, and 2016. For example, to compute an estimate for 1964, the 1960 value was subtracted from the 1970 value, the difference was divided by nine, and four times this difference was added to the value for 1960 to produce an estimated value for 1964.

#### Urban Population Percent

The urban population percent for each state was obtained for 1960, 1970, 1980, 1990, 2000, and 2010 from ISU (2022). The state urbanization scores produced by Rakich (2020) were used for 2020. Prorated urban population percent estimates were created using the procedure for White population percent. However, the prorated estimates for 2012 and 2016 were based on standardized urban population percent values for 2010 and 2020 because the development of the 2020 variable followed a unique procedure and yielded values not directly comparable to urban population percent values for the years from 1960 to 2010.

### Analytic Strategy

Planned Pearson correlations, partial correlations, and multiple regressions were used to determine the relation between the state citizen ideology scores and handedness without and with the consideration of Big Five personality, income, White population percent, and urbanization as covariates for each of the presidential election years from 1964 to 2016. The same procedures were employed to determine the relation between the Democratic-Republican percent of the popular vote and handedness for each of the presidential elections from 1964 to 2020. As well, similar procedures were used to determine the relation of state citizen ideology to state Democratic-Republican percent of the popular vote when handedness, the Big Five, income, race, and urbanization were considered as statistical controls. Two-tailed significance tests and an alpha level of .05 were used throughout.

## Results

Table 1 displays the means and standard deviations for handedness, Big Five personality, income, White population percent, and urban population percent for each of the presidential election years from 1964 to 2020.

**Table 1.** Means and Standard Deviations for Handedness, Big Five Personality, Income, White Population Percent, and Urbanization for 1964–2020.

Variable	*M*	*SD*	Variable	*M*	*SD*	Variable	*M*	*SD*
Handedness	11.64	.94	Income 2008	39,959.44	6094.48	White 2016	70.68	13.09
Openness	−.01	.87	Income 2012	43,607.00	6796.95	White 2020	69.66	12.69
Conscientiousness	.11	.89	Income 2016	47,823.90	7117.52	Urban 1964	63.70	14.56
Extraversion	.01	.97	Income 2020	57,352.88	8396.75	Urban 1968	65.46	14.41
Agreeableness	.13	.74	White 1964	89.78	9.56	Urban 1972	66.06	14.36
Neuroticism	.05	1.01	White 1968	89.62	8.99	Urban 1976	66.36	14.37
Income 1964	2542.25	452.10	White 1972	88.95	8.80	Urban 1980	66.59	14.42
Income 1968	3351.54	548.54	White 1976	87.70	8.88	Urban 1984	67.11	14.51
Income 1972	4603.98	632.17	White 1980	86.75	9.09	Urban 1988	67.63	14.63
Income 1976	6548.65	799.24	White 1984	86.01	9.19	Urban 1992	68.57	14.65
Income 1980	9683.90	1298.06	White 1988	85.27	9.34	Urban 1996	70.19	14.74
Income 1984	13,187.54	1777.93	White 1992	84.14	9.56	Urban 2000	71.41	14.91
Income 1988	16,418.71	2695.71	White 1996	82.25	9.96	Urban 2004	72.27	14.75
Income 1992	20,057.75	2857.90	White 2000	80.84	10.31	Urban 2008	73.14	14.61
Income 1996	23,733.94	3216.11	White 2004	77.23	11.76	Urban 2012	.00	.98
Income 2000	29,212.56	4724.61	White 2008	73.62	13.59	Urban 2016	.00	.98
Income 2004	33,114.25	4891.59	White 2012	72.04	13.73	Urban 2020	10.43	1.06

*p < .05. **p < .01. ***p < .001. All tests are two-tailed.

Table 2 presents the Pearson correlations of state handedness to each of those variables for the same years. Of course, as in the McCann (2018a) research, state left-handedness was associated significantly with higher openness to experience (.49), lower conscientiousness (−.45), lower extraversion (−.32), lower agreeableness (−.38), and higher neuroticism (.39). Left-handedness also was significantly associated with higher income in all years from 1964 to 2020 with correlations ranging from .34 in 2012 to .65 in 1988. However, handedness was only significantly associated with higher levels of urbanization in 1964 (.29), 2016 (.31), and 2020 (.38). Handedness was not correlated significantly with White population percent for any of the years.

**Table 2.** Pearson Correlations of Handedness With Big Five Personality, Income, White Population Percent, and Urbanization for 1964–2020.

Variable	*r*	Variable	*r*	Variable	*r*
Openness	.49***	Income 2012	.34*	White 2020	−.06
Conscientiousness	−.45***	Income 2016	.46***	Urban 1964	.29*
Extraversion	−.32*	Income 2020	.44**	Urban 1968	.25
Agreeableness	−.38**	White 1964	.20	Urban 1972	.22
Neuroticism	.39**	White 1968	.18	Urban 1976	.19
Income 1964	.49***	White 1972	.17	Urban 1980	.17
Income 1968	.53***	White 1976	.15	Urban 1984	.15
Income 1972	.43**	White 1980	.14	Urban 1988	.13
Income 1976	.35*	White 1984	.12	Urban 1992	.14
Income 1980	.37**	White 1988	.09	Urban 1996	.16
Income 1984	.49***	White 1992	.08	Urban 2000	.18
Income 1988	.65***	White 1996	.05	Urban 2004	.17
Income 1992	.60***	White 2000	.04	Urban 2008	.15
Income 1996	.57***	White 2004	.02	Urban 2012	.20
Income 2000	.58***	White 2008	−.00	Urban 2016	.31*
Income 2004	.60***	White 2012	−.02	Urban 2020	.38**
Income 2008	.44**	White 2016	−.04		

*p < .05. **p < .01. ***p < .001. All tests are two-tailed.

Curiously, correlations were significant and positive between handedness and income for each of the election years from 1964 to 2016 in Table 2. In an exploratory fashion, partial correlations were computed using variables available in the current database to perhaps find an explanation for the relations between handedness and income. Because there also was often a positive correlation between income and political preference, the first strategy was to control simultaneously for ideology and Democratic-Republican popular vote for president. These partial correlations reduced the relation between handedness and income to non-significance for 1964, 1972, 1976, 1980, 2000, 2004, 2008, 2012, 2016, and 2020. Because Big Five personality variables also often showed significant correlations with income and all showed correlations with handedness, the Big Five were added to the control pool for 1968, 1984, 1988, 1992 and 1996. This strategy reduced the relation between handedness and income to non-significance for 1968, 1984, and 1996. Furthermore, although still significant, the correlation between handedness and income was reduced from .65 to .36 for 1988 and from .60 to .32 for 1992. Therefore, it is evident from the present data that political preference and the Big Five—which are both related to handedness and income—may play a prominent role in the correlation between handedness and income.

Table 3 shows the correlation between state Conservative-Liberal ideology and Democratic-Republican popular vote for each of the presidential election years from 1964 to 2016. Correlations ranged from −.00 in 1976 to .88 in 2016 (*M* = 67.5, *SD* = 25.1). The four highest correlations occurred for 2004, 2008, 2012, and 2016.

**Table 3.** Pearson Correlations Between Conservative-Liberal Ideology and Democratic-Republican Popular Vote for Each of the Presidential Election Years 1964–2016.

Year	*r*	Year	*r*	Year	*r*
1964	.79***	1984	.67***	2004	.87***
1968	.40**	1988	.74***	2008	.85***
1972	.84***	1992	.63***	2012	.86***
1976	−.00	1996	.76***	2016	.88***
1980	.39**	2000	.77***		

*p < .05. **p < .01. ***p < .001. All tests are two-tailed.

### Conservative-Liberal Ideology Relations

Table 4 presents means and standard deviations for state Conservative-Liberal ideology for each of the presidential election years from 1964 to 2016. Table 4 also shows the relations between handedness and the political ideology variable based on Pearson correlations and five partial correlations for each election. Partial correlation A adjusted for state Big Five personality variables, partial correlation B for income, partial correlation C for White population percent, and partial correlation D for urban population percent. Partial correlation E adjusted jointly for Big Five, income, White population percent, and urban population percent.

**Table 4.** Means, Standard Deviations, and Pearson and Partial Correlations of Handedness With Conservative-Liberal Ideology 1964–2016.

Variable	*M*	*SD*	Winner	Pearson	Partial A^a^	Partial B^b^	Partial C^c^	Partial D^d^	Partial E^e^
1964 Conservative-Liberal ideology	50.10	16.96	Democrat	.54***	.37*	.39**	.54***	.49***	.31
1968 Conservative-Liberal ideology	41.76	21.43	Republican	.53***	.29	.28	.54***	.48***	.06
1972 Conservative-Liberal ideology	40.88	17.48	Republican	.57***	.45**	.45***	.61***	.54***	.39*
1976 Conservative-Liberal ideology	44.53	15.29	Democrat	.62***	.41**	.55***	.65***	.61***	.33*
1980 Conservative-Liberal ideology	42.14	16.15	Republican	.56***	.33*	.48***	.56***	.55***	.24
1984 Conservative-Liberal ideology	45.22	15.34	Republican	.56***	.36*	.45**	.56***	.55***	.26
1988 Conservative-Liberal ideology	56.09	15.38	Republican	.62***	.42**	.46***	.62***	.62***	.29
1992 Conservative-Liberal ideology	53.84	11.77	Democrat	.58***	.37*	.41**	.59***	.58***	.18
1996 Conservative-Liberal ideology	44.47	13.54	Democrat	.58***	.34*	.39**	.58***	.56***	.18
2000 Conservative-Liberal ideology	43.22	14.45	Republican	.64***	.41**	.47***	.64***	.62***	.35*
2004 Conservative-Liberal ideology	51.70	14.99	Republican	.71***	.53***	.56***	.71***	.70***	.42**
2008 Conservative-Liberal ideology	61.14	15.85	Democrat	.77***	.62***	.72***	.77***	.77***	.59***
2012 Conservative-Liberal ideology	47.89	14.83	Democrat	.74***	.56***	.70***	.74***	.73***	.54***
2016 Conservative-Liberal ideology	51.84	16.08	Republican	.80***	.64***	.74***	.81***	.78***	.64***

^a^Partial correlation A controls for the Big Five personality variables.

^b^Partial correlation B controls for income.

^c^Partial correlation C controls for White population percent.

^d^Partial correlation D controls for urbanization.

^e^Partial correlation E controls for Big Five personality, income, White population percent, and urbanization.

*p < .05. **p < .01. ***p < .001. All tests are two-tailed.

The Pearson correlations showed that state left-handedness was associated significantly with greater state liberal ideology in each of the 14 elections from 1964 to 2016. The significant positive correlation between handedness and ideology occurred in the six election years won by a Democrat and the eight won by a Republican. Correlations ranged from .53 to .80 (*M* = .63, *SD* = .09) for the 14 elections, from .54 to .77 (*M* = .64, *SD* = .09) for the six years with Democratic winners, and from .53 to .80 (*M* = .62, *SD* = .09) for the eight years with Republican winners.

Most of the partial correlations also strongly supported the association between left-handedness and state tendencies to endorse a more liberal ideology. With either Big Five personality or income controlled, the correlation was positive and significant for all election years except 1968. With either White or urban population percent controlled, the correlation was positive and significant for each of the 14 election years. With the Big Five, income, White population percent, and urban population percent jointly controlled, the correlation was positive and significant only for 1972, 1976, 2000, 2004, 2008, 2012, and 2016, although the sign was positive for all years.

The partial correlations in Table 4 describe the nature and degree of relation between the citizen ideology variable and handedness with the variance explained by the covariate or covariates extracted from both the ideology variable *and* handedness. In other words, the partial correlation is an *adjusted* correlation. However, another way to examine the relation between ideology and handedness is to use a multiple regression strategy with the ideology variable as the criterion and handedness and the other covariates as predictors potentially accounting for variance in the criterion. With this approach, the variance explained by the covariate or covariates is extracted only from the ideology variable.

In the present regression model, handedness was forced to enter the equation on the first step and was essentially controlled. On the second step, forward stepwise selection was used to choose statistically nonredundant predictors for entry into the equation from the pool of eight potential predictors—the Big Five and income, White, and urbanization variables. Therefore, the cumulative variance in the criterion that could be accounted for by handedness and each of the additional nonredundant predictors could be assessed, and the independence and relative magnitude of contribution of handedness and each of the other predictors in the full equation could be gauged by the standardized regression coefficients (βs) for each of the election years from 1964 to 2016. The construction process for these two-step multiple regression equations afforded the possibility of results that differed substantively from those of the partial correlations.

The multiple regression results for ideology are displayed in Table 5. As could be deduced from the Pearson correlations in Table 4, with handedness entered first in the equations, it accounted for significant variance in ideology for all election years, ranging from 27.8% in 1968 to 64.4% in 2016 (*M* = 40.4, *SD* = 11.8). Except for 1968, for each of the full equations including the other selected predictors, handedness also surfaced as an independent predictor with positive βs ranging from .34 to .77 (*M* = .54, *SD* = .15).

**Table 5.** Multiple Regression Equations Demonstrating the Relation of Handedness, Big Five Personality, Income, White Population Percent, and Urbanization to Conservative-Liberal Ideology 1964–2016.

Year	Step	Entry	Predictor Pool	Stepwise Predictors	df	*R*^2^ Change	*F*	Significant Predictors	β	*t*
1964	1	Forced	Handedness		1, 46	.292	18.96***	White percent 1964	.50	5.05***
	2	Stepwise	Big five	Conscientiousness	1, 45	.074	5.24*	Handedness	.35	3.28**
	3	Stepwise	Pool of three^a^	White percent 1964	1, 44	.233	25.47*			
1968	1	Forced	Handedness		1, 46	.278	17.68***	White percent 1968	.43	4.68***
	2	Stepwise	Big five	Conscientiousness	1, 45	.100	7.22**	Income 1968	.34	3.27**
	3	Stepwise	Pool of three	White percent 1968	1, 44	.236	26.89***	Conscientiousness	−.22	−2.23*
				Income 1968	1, 43	.077	10.69**			
1972	1	Forced	Handedness		1, 46	.329	22.60***	White percent 1972	.56	6.36***
	2	Stepwise	Big five	(None)				Handedness	.43	4.81***
	3	Stepwise	Pool of three	White percent 1972	1, 45	.294	35.14***	Urban percent 1972	.23	2.58*
				Urban percent 1972	1, 44	.049	6.65*			
1976	1	Forced	Handedness		1, 46	.388	29.19***	Handedness	.44	4.36***
	2	Stepwise	Big five	Conscientiousness	1, 45	.129	12.01***	White percent 1976	.38	3.99***
	3	Stepwise	Pool of three	White percent 1976	1, 44	.128	15.95***	Conscientiousness	−.29	−2.77**
1980	1	Forced	Handedness		1, 46	.315	21.14***	Conscientiousness	−.60	−4.77***
	2	Stepwise	Big five	Conscientiousness	1, 45	.161	13.88***	Agreeableness	.41	3.21**
				Agreeableness	1, 44	.059	5.62*	Handedness	.34	3.02**
	3	Stepwise	Pool of three	Income 1980	1, 43	.067	7.20**	Income 1980	.30	2.68**
1984	1	Forced	Handedness		1, 46	.314	21.09***	Handedness	.56	4.59***
	2	Stepwise	Big five	(None)						
	3	Stepwise	Pool of three	(None)						
1988	1	Forced	Handedness		1, 46	.384	28.67***	Handedness	.54	4.59***
	2	Stepwise	Big five	Conscientiousness	1, 45	.069	5.64*	Conscientiousness	−.48	−3.54***
				Agreeableness	1, 44	.076	7.12*	Agreeableness	.35	2.67*
	3	Stepwise	Pool of three	(None)						
1992	1	Forced	Handedness		1, 46	.333	22.96***	Handedness	.47	3.76***
	2	Stepwise	Big five	Conscientiousness	1, 45	.064	4.81*	White percent 1992	.27	2.31*
	3	Stepwise	Pool of three	White percent 1992	1, 44	.065	5.33*			
1996	1	Forced	Handedness		1, 46	.332	22.90***	Handedness	.40	2.84**
	2	Stepwise	Big five	(None)				Income 1996	.31	2.19*
	3	Stepwise	Pool of three	Income 1996	1, 45	.064	4.77*			
2000	1	forced	Handedness		1, 46	.405	31.37***	Handedness	.64	5.60***
	2	Stepwise	Big five	(None)						
	3	Stepwise	Pool of three	(None)						
2004	1	Forced	Handedness		1, 46	.505	46.86***	Handedness	.71	6.85***
	2	Stepwise	Big five	(None)						
	3	Stepwise	Pool of three	(None)						
2008	1	Forced	Handedness		1, 46	.587	65.50***	Handedness	.77	8.09***
	2	Stepwise	Big five	(None)						
	3	Stepwise	Pool of three	(None)						
2012	1	Forced	Handedness		1, 46	.543	54.67***	Handedness	.74	7.39***
	2	Stepwise	Big five	(None)						
	3	Stepwise	Pool of three	(None)						
2016	1	Forced	Handedness		1, 46	.644	83.33***	Handedness	.67	7.69***
	2	Stepwise	Big five	(None)				Income 2016	.27	3.03**
	3	Stepwise	Pool of three	Income 2016	1, 45	.063	9.62**	White percent 2016	−.16	−2.06*
				White percent 2016	1, 44	.026	4.25*			

^a^The pool of three potential predictors included the year-appropriate income, urbanization, and White percent variables.

*p < .05. **p < .01. ***p < .001. All tests are two-tailed.

Among the pool of the other eight potential predictors of Conservative-Liberal ideology, only conscientiousness, agreeableness, income, White population percent, and urbanization ever accounted for criterion variance with handedness and perhaps other variables controlled. Conscientiousness did so in 1964, 1968, 1976, 1980, 1988, 1992, and 2008. Agreeableness did so in 1980 and 1988. Income did so in 1968, 1980, 1996, and 2016. White population percent did so in 1964, 1968, 1972, 1976, 1992, and 2016. Urbanization did so only in 1972. Significant negative β coefficients emerged for conscientiousness in 1968, 1976, 1980, and 1988. Significant positive βs occurred for agreeableness in 1980 and 1988. Significant positive βs surfaced for income in 1968, 1980, 1996, and 2016. Significant positive βs emerged for White population percent in 1964, 1968, 1972, 1976, and 1992, but a significant negative β emerged in 2016. The β also was significant and positive for urbanization in 1972. The significant βs, and their mean and standard deviation regardless of sign, ranged from .22 to .60 (*M* = .40, *SD* = .17) for conscientiousness, from .35 to .41 (*M* = .38, *SD* = .04) for agreeableness, from .27 to .34 (*M* = .31, *SD* = .03) for income, and from −.16 to .56 (*M* = .38, *SD* = .19) for White population percent. The lone significant β for urbanization was .23.

### Democratic-Republican Popular Vote Relations

Table 6 displays means and standard deviations for state Democratic-Republican popular vote for president in each election year from 1964 to 2020. Table 6 also contains the relations between handedness and the popular vote variable based on Pearson correlations and the five partial correlations for each election. The Pearson correlations showed that state left-handedness was associated significantly with higher Democratic-Republican popular vote in 14 of the 15 presidential elections from 1964 to 2020. The significant positive correlation between handedness and Democratic-Republican popular vote occurred in six of the seven elections won by a Democrat and the eight won by a Republican. Correlations ranged from .18 to .79 (*M* = .59, *SD* = .18) for the 15 elections, from .18 to .77 (*M* = .62, *SD* = .21) for the seven years with Democratic winners, and from .38 to .79 (*M* = .56, *SD* = .17) for the eight years with Republican winners.

**Table 6.** Means, Standard Deviations, and Pearson and Partial Correlations of Handedness With Democratic-Republican Popular Vote for Presidential Candidates 1964–2020.

Variable	*M*	*SD*	Winner	Pearson	Partial A^a^	Partial B^b^	Partial C^c^	Partial D^d^	Partial E^e^
1964 Democratic-Republican vote	16.76	21.87	Democrat	.57***	.41**	.40**	.63***	.52***	.35*
1968 Democratic-Republican vote	−4.21	12.14	Republican	.46***	.08	.42**	.50***	.43**	.22
1972 Democratic-Republican vote	−27.50	13.48	Republican	.51***	.39**	.37**	.51***	.48***	.29
1976 Democratic-Republican vote	.13	11.95	Democrat	.18	−.00	.31*	.28	.21	.14
1980 Democratic-Republican vote	−12.96	14.55	Republican	.38**	.16	.45**	.46***	.40**	.26
1984 Democratic-Republican vote	−22.12	11.44	Republican	.46***	.23	.41**	.47***	.46***	.24
1988 Democratic-Republican vote	−10.26	10.82	Republican	.40**	.20	.28	.39**	.39**	.17
1992 Democratic-Republican vote	2.71	9.39	Democrat	.61***	.32*	.51***	.62***	.61***	.30
1996 Democratic-Republican vote	5.43	13.20	Democrat	.74***	.52***	.65***	.74***	.73***	.51***
2000 Democratic-Republican vote	−5.16	16.78	Republican	.77***	.58***	.64***	.80***	.78***	.58***
2004 Democratic-Republican vote	−7.43	16.92	Republican	.79***	.61***	.67***	.80***	.79***	.52***
2008 Democratic-Republican vote	2.27	17.96	Democrat	.77***	.64***	.71***	.77***	.77***	.63***
2012 Democratic-Republican vote	−2.31	19.78	Democrat	.76***	.61***	.73***	.78***	.76***	.64***
2016 Democratic-Republican vote	−6.05	19.80	Republican	.69***	.54***	.59***	.74***	.68***	.60***
2020 Democratic-Republican vote	−2.94	20.63	Democrat	.70***	.56***	.61***	.68***	.64***	.59***

^a^Partial correlation A controls for the Big Five personality variables.

^b^Partial correlation B controls for income.

^c^Partial correlation C controls for White population percent.

^d^Partial correlation D controls for urban population percent.

^e^Partial correlation E controls for Big Five personality, income, White population percent, and urbanization.

*p < .05. **p < .01. ***p < .001. All tests are two-tailed.

Most of the partial correlations also strongly supported the association between left-handedness and state tendencies to vote for a Democratic presidential candidate. With income controlled, the correlation was positive and significant for all election years except 1988. With either White or urban population percent controlled, the correlation was positive and significant for all elections except 1976. With the Big Five controlled, the correlation was positive and significant for 1964, 1972, 1992, and each election afterward. With all eight controls in place, the correlation was positive and significant for 1964, 1996, and each following election. However, the sign of each of the nonsignificant partial correlations in Table 6 was positive except for the sole exception of −.00 in 1976 with the Big Five serving as the statistical controls.

The multiple regression results for Democratic-Republican popular vote are presented in Table 7. As could be deduced from the Pearson correlations in Table 6 excluding 1976, handedness accounted for significant variance in the Democratic-Republican criterion ranging from 14.4% in 1980 to 62.9% in 2004 (*M* = 40.0, *SD* = 17.9). Except for 1968, 1976, and 1980, for each of the full equations including the other selected predictors, handedness emerged as an independent predictor with positive βs ranging from .34 to .73 (*M* = .52, *SD* = .15).

**Table 7.** Multiple Regression Equations Demonstrating the Relation of Handedness, Big Five Personality, Income, White Population Percent, and Urbanization to Democratic-Republican Presidential Vote 1964–2020.

Year	Step	Entry	Predictor Pool	Stepwise Predictors	df	*R*^2^ Change	*F*	Significant Predictors	β	*t*
1964	1	Forced	Handedness		1, 46	.323	21.99***	White percent 1964	.62	7.89***
	2	Stepwise	Big five	(None)				Handedness	.40	4.87***
	3	Stepwise	Pool of three^a^	White percent 1964	1, 45	.389	60.93*	Urban percent 1964	.17	2.04*
				Urban percent 1964	1, 44	.025	4.15*			
1968	1	Forced	Handedness		1, 46	.215	12.58***	Neuroticism	.49	3.87***
	2	Stepwise	Big five	Neuroticism	1, 45	.162	11.67***	Urban percent 1968	.24	2.27*
	3	Stepwise	Pool of three	Urban percent 1968	1, 44	.053	4.11*			
1972	1	Forced	Handedness		1, 46	.261	16.21***	White percent 1972	.48	4.57***
	2	Stepwise	Big five	(None)				Handedness	.38	3.53***
	3	Stepwise	Pool of three	White percent 1972	1, 45	.218	18.77***	Urban percent 1972	.23	2.19*
				Urban percent 1972	1, 44	.051	4.80*			
1976	1	Forced	Handedness		1, 46	.031	1.49	White percent 1976	−.41	−3.22**
	2	Stepwise	Big five	Neuroticism	1, 45	.194	11.27**	Neuroticism	.35	2.60*
	3	Stepwise	Pool of three	White percent 1976	1, 44	.148	10.38**			
1980	1	Forced	Handedness		1, 46	.144	7.73**	Neuroticism	.47	3.92***
	2	Stepwise	Big five	Neuroticism	1, 45	.261	19.69***	White percent 1980	−.30	2.70**
	3	Stepwise	Pool of three	White percent 1980	1, 44	.085	7.28**			
1984	1	Forced	Handedness		1, 46	.215	12.57***	Handedness	.34	2.54*
	2	Stepwise	Big five	Neuroticism	1, 45	.081	5.19*	Neuroticism	.31	2.28*
	3	Stepwise	Pool of three	(None)						
1988	1	Forced	Handedness		1, 46	.156	8.48**	Handedness	.40	2.91**
	2	Stepwise	Big five	(None)						
	3	Stepwise	Pool of three	(None)						
1992	1	Forced	Handedness		1, 46	.376	27.66***	Handedness	.52	4.23***
	2	Stepwise	Big five	Neuroticism	1, 45	.053	4.13*	Neuroticism	.25	2.03*
	3	Stepwise	Pool of three	(None)						
1996	1	Forced	Handedness		1, 46	.548	55.86***	Handedness	.63	6.31***
	2	Stepwise	Big five	Neuroticism	1, 45	.071	8.35**	Neuroticism	.29	2.89**
	3	Stepwise	Pool of three	(None)						
2000	1	Forced	Handedness		1, 46	.598	68.43***	Handedness	.72	8.32***
	2	Stepwise	Big five	(None)				Urban percent 2000	.28	3.17**
	3	Stepwise	Pool of three	Urban percent 2000	1, 45	.073	10.05**			
2004	1	Forced	Handedness		1, 46	.629	78.11***	Handedness	.65	6.00***
	2	Stepwise	Big five	(None)				Income 2004	.24	2.27*
	3	Stepwise	Pool of three	Income 2004	1, 45	.038	5.13*			
2008	1	Forced	Handedness		1, 46	.588	65.66***	Handedness	.73	8.08***
	2	Stepwise	Big five	(None)				Urban percent 2008	.23	2.48*
	3	Stepwise	Pool of three	Urban percent 2008	1, 45	.049	6.15*			
2012	1	Forced	Handedness		1, 46	.584	64.53***	Handedness	.72	7.84***
	2	Stepwise	Big five	(None)				Urban percent 2012	.24	2.65*
	3	Stepwise	Pool of three	Urban percent 2012	1, 45	.056	7.00*			
2016	1	Forced	Handedness		1, 46	.476	41.78***	Handedness	.47	5.05***
	2	Stepwise	Big five	Openness	1, 45	.119	13.21***	Urban percent 2016	.41	4.28***
	3	Stepwise	Pool of three	Urban percent 2016	1, 44	.119	18.33***			
2020	1	Forced	Handedness		1, 46	.489	44.04***	Handedness	.40	4.09***
	2	Stepwise	Big five	Openness	1, 45	.099	10.85**	Urban percent 2020	.27	2.55*
	3	Stepwise	Pool of three	Urban percent 2020	1, 44	.092	12.71***	Income 2020	.23	2.34*
				Income 2020	1, 44	.036	5.45*			

^a^The pool of nine potential predictors included the Big Five and year-appropriate income, urbanization, and White percent variables.

*p < .05. **p < .01. ***p < .001. All tests are two-tailed.

Among the pool of the other eight potential predictors of Democratic-Republican popular vote, only neuroticism, openness to experience, income, White population percent, and urbanization ever accounted for significant additional criterion variance. Neuroticism did so in 1968, 1976, 1980, 1984, 1992, and 1996. Openness did in 2016 and 2020. Income did in 2004 and 2020. White population percent did in 1964, 1972, 1976, and 1980. Urbanization did in 1964, 1968, 1972, 2000, 2008, 2012, 2016, and 2020. Significant positive β coefficients emerged for neuroticism in 1968, 1976, 1980, 1984, 1992, and 1996. No significant βs occurred for openness. Significant positive βs surfaced for income in 2004 and 2020. Significant positive βs emerged for White population percent in 1964 and 1972, but significant negative βs emerged in 1976 and 1980. The β also was significant and positive for urbanization in 1972. The significant βs, and their mean and standard deviation regardless of sign, ranged from .22 to .60 (*M* = .40, *SD* = .17) for conscientiousness, from .35 to .41 (*M* = .38, *SD* = .04) for agreeableness, from .27 to .34 (*M* = .31, *SD* = .03) for income, and from −.16 to .56 (*M* = .38, *SD* = .19) for White population percent. The sole significant β for urbanization was 23.

### Relations of Conservative-Liberal Ideology to Democratic-Republican Popular Vote With the Significant Predictors From Table 7 Serving as Statistical Controls

Table 8 displays the results of multiple regression equations in which the predictors of Democratic-Republican popular vote with significant βs in the corresponding equations of Table 7 were entered as a block and followed by the *stepwise* entry of the Conservative-Liberal ideology variable. Such stepwise entry ensured that the ideology variable would only enter the equation if it could account for significant additional variance in the popular vote criterion.

**Table 8.** Multiple Regression Equations Demonstrating the Relation of Conservative-Liberal Ideology to Democratic-Republican Presidential Vote 1964-2020 When Appended to the Predictors With Significant βs in the Equations of Table 7.

Year	Step	Entry	Predictor Pool	Stepwise Predictors	df	*R*^2^ change	*F*	Significant Predictors	β	*t*
1964	1	Forced	Handedness		3, 44	.737	41.17***	White percent 1964	.45	4.82***
			White percent 1964					Ideology 1964	.33	2.92**
			Urbanization 1964					Handedness	.27	3.10**
	2	Stepwise	Ideology 1964	Ideology 1964	1, 43	.044	8.55**			
1968	1	Forced	Handedness		3, 44	.430	11.05***	Neuroticism	.49	3.87***
			Neuroticism					Urbanization 1968	.24	2.03*
			Urbanization 1968							
	2	Stepwise	Ideology 1968	(None)						
1972	1	Forced	Handedness		3, 44	.530	16.51***	Ideology 1972	.75	5.26***
			White percent 1972							
			Urbanization 1972							
	2	Stepwise	Ideology 1972	Ideology 1972	1, 43	.184	27.64***			
1976	1	Forced	Handedness		3, 44	.373	8.74***	White percent 1976	−.41	−3.22**
			Neuroticism					Neuroticism	.35	2.60*
			White percent 1976							
	3	Stepwise	Ideology 1976	(None)						
1980	1	Forced	Handedness		3, 44	.489	14.04**	White percent 1980	−.48	−4.16***
			Neuroticism					Ideology 1980	.44	3.30**
			White percent 1980					Neuroticism	.38	3.43***
	2	Stepwise	Ideology 1980	Ideology 1980	1, 43	.103	10.87**			
1984	1	Forced	Handedness		2, 45	.296	9.46***	Ideology 1984	.56	4.32***
			Neuroticism					Neuroticism	.25	2.12*
	2	Stepwise	Ideology 1984	Ideology 1984	1, 43	.210	18.69***			
1988	1	forced	Handedness		1, 46	.154	8.34**	Ideology 1988	.83	6.70***
	2	Stepwise	Ideology 1988	Ideology 1988	1, 45	.422	44.83***			
1992	1	Forced	Handedness		2, 45	.428	16.84***	Ideology 1992	.38	2.94**
			Neuroticism					Handedness	.31	2.38*
	2	Stepwise	Ideology 1992	Ideology 1992	1, 44	.094	8.65**			
1996	1	Forced	Handedness		2, 45	.619	36.57***	Ideology 1996	.47	5.20***
			Neuroticism					Handedness	.37	3.99***
	2	Stepwise	Ideology 1996	Ideology 1996	1, 44	.145	27.02***	Neuroticism	.25	3.18**
2000	1	Forced	Handedness		2, 45	.671	45.97***	Handedness	.48	5.03***
			Urbanization 2000					Ideology 2000	.41	4.23***
	2	Stepwise	Ideology 2000	Ideology 2000	1, 44	.095	17.89***	Urbanization 2000	.21	2.70**
2004	1	Forced	Handedness		2, 45	.667	45.13***	Ideology 2004	.59	6.38***
			Income 2004					Handedness	.31	3.21**
	2	Stepwise	Ideology 2004	Ideology 2004	1, 44	.160	40.76***			
2008	1	Forced	Handedness		2, 45	.638	39.57***	Ideology 2008	.66	6.54***
			Urbanization 2008					Urbanization 2008	.26	3.98***
	2	Stepwise	Ideology 2008	Ideology 2008	1, 44	.179	42.70***	Handedness	.22	2.17*
2012	1	Forced	Handedness		2, 45	.640	39.98***	Ideology 2012	.66	7.37***
			Urbanization 2012					Urbanization 2012	.24	3.94***
	2	Stepwise	Ideology 2012	Ideology 2012	1, 44	.199	54.36***	Handedness	.23	2.53*
2016	1	Forced	Handedness		3, 44	.714	36.62***	Ideology 2016	.70	6.73***
			Openness					Urbanization 2016	.26	3.72***
			Urbanization 2016					Openness to experience	.15	2.02*
	2	Stepwise	Ideology 2016	Ideology 2016	1, 43	.147	45.32***			

*p < .05. **p < .01. ***p < .001. All tests are two-tailed.

Conservative-Liberal ideology still accounted for further variance in the Democratic-Republican criterion in each of the elections from 1964 to 2016, except in 1968 and 1976. The significant increment in variance accounted for in the other 12 elections ranged from 4.4% to 42.2% (*M* = 16.5, *SD* = .09). Significant βs also emerged for each of the years that ideology accounted for additional criterion variance, with values ranging from .33 to .83 (*M* = .57, *SD* = .16).

Although handedness which entered the equations first and always accounted for significant variance in Table 6 except for 1976, it only surfaced as an independent predictor in the full equations of Table 8 for 1964, 1992, 1996, 2000, 2004, 2008, and 2012. The significant βs for these years ranged from .22 to .48 (*M* = .31, *SD* = .09).

Among the other potential predictors, neuroticism was an independent predictor in 1968, 1976, 1980, 1984, and 1996 with significant βs ranging from .25 to .49 (*M* = .34, *SD* = .10). Openness to experience also produced a significant β of .15 in 2016. As well, White population percent yielded significant βs of .45 in 1964, −.41 in 1976, and −.48 in 1980. Urban population percent was an independent predictor in 1968, 2000, 2008, 2012, and 2016 with significant βs ranging from .21 to .26 (*M* = .24, *SD* = .02).

A summary of the independent predictors—those with significant β coefficients—in Table 5, Table 7, and Table 8 is presented in Table 9. The predominant role of handedness as a predictor of ideology and party preference is clear. The continuing predictive capacity of ideology in relation to political party preference even with the consideration of the other predictors also is evident.

**Table 9.** A Summary of the Independent Predictors (As Indicated by Significant Regression Coefficients) of Ideology in Table 5 and Political Party Preference in Table 7 and Table 8.

Criterion	Table	Independent Predictor	1964	1968	1972	1976	1980	1984	1988	1992	1996	2000	2004	2008	2012	2016	2020
Ideology	4	Handedness	Yes		Yes	Yes	Yes	Yes	Yes	Yes	Yes	Yes	Yes	Yes	Yes	Yes	N/A
		White percent	Yes	Yes	Yes	Yes				Yes						Yes	N/A
		Urbanization			Yes												N/A
		Income		Yes			Yes				Yes					Yes	N/A
		Conscientiousness		Yes		Yes	Yes		Yes								N/A
		Agreeableness					Yes		Yes								N/A
																	
Party preference	6	Handedness	Yes		Yes		Yes	Yes	Yes	Yes	Yes	Yes	Yes	Yes	Yes	Yes	Yes
		White percent	Yes		Yes	Yes	Yes										
		Urbanization	Yes	Yes	Yes							Yes		Yes	Yes	Yes	Yes
		Income											Yes				Yes
		Neuroticism		Yes		Yes	Yes	Yes		Yes	Yes						
																	
Party preference	7	Handedness	Yes							Yes	Yes	Yes	Yes	Yes	Yes		N/A
		White percent	Yes			Yes	Yes										N/A
		Urbanization		Yes								Yes		Yes	Yes	Yes	N/A
		Neuroticism		Yes		Yes	Yes	Yes			Yes						N/A
		Openness														Yes	N/A
		Ideology	Yes		Yes		Yes	Yes	Yes	Yes	Yes	Yes	Yes	Yes	Yes	Yes	N/A

### Spatial Autocorrelation in the Equations of Table 5, Table 7, and Table 8

The multiple regression equations in Table 5, Table 7, and Table 8 were tested for the presence of spatial autocorrelation with the global Moran’s I test (Anselin, 2003) and its potential remedy was addressed where necessary with spatial lag multiple regression equations using the R *haven, spdep,* and *spatialreg* packages (R, 2022). To facilitate the spatial autocorrelation analyses in R, all the relevant variables were converted to z score form. A Queen’s binary *neighborhood* matrix also was created which treated each state that touched another state at any point as a neighbor. For each of the 43 original regression equations, the spatial autocorrelation test was conducted on a simultaneous equation that contained all the predictors that had entered the original equation.

The spatial autocorrelation results are shown in Table 10. Significant Moran’s I statistic standard deviates indicated that equations from Table 5 for 1972, 1976, 1980, and 1984, from Table 7 for 1964, 1972, 1976, 1980, 1984, and 1988, and from Table 8 for 1964, 1976, 1980, 1984, 2000, 2008, and 2016 showed evidence of spatial autocorrelation. However, when spatial lag regression equations controlling for such autocorrelation were created that included the significant independent predictors in the original regression equations, the significant βs of the lag equations generally reflected those of the original equations. Signs remained the same and the lag regression βs were still significant for all predictors except handedness in the 1980 equation in Table 5, neuroticism in the 1984 equation in Table 7, and neuroticism in the 1984 equation in Table 8. These three βs approached significance in the lag equations with probability levels of .084, .088. and .097, respectively, and each would have been significant with a rather defensible one-tailed test if it had been chosen. The only lag regression equation to show any sign of remaining residual autocorrelation was the one for 2016 from Table 8 with an LM test value of 4.41 significant at the .05 level. Overall, the spatial lag regression equation results do not differ substantively from the original results. Therefore, interpretation can be based justifiably on the original least squares multiple regression solutions despite the spatial autocorrelation that was detected for 17 of the 43 original multiple regression equations from Table 5, Table 7, and Table 8.

**Table 10.** Significant Spatial Autocorrelation Results for Multiple Regression Equations in Table 5, Table 7, and Table 8.

Criterion	Table	Moran’s I^a^	Original Regression Equation		Spatial Lag Regression Equation		
			Significant Predictors	Significant βs	Significant Predictors	Significant βs	LM test^b^
1972 Conservative-Liberal	4	2.42**	Handedness	.43***	Handedness	.31***	2.85
			White percent 1972	.56***	White percent 1972	.37***	
			Urbanization 1972	.23*	Urbanization 1972	.17*	
1976 Conservative-Liberal	4	2.02*	Handedness	.44***	Handedness	.29**	1.17
			Conscientiousness	−.29**	Conscientiousness	−.19*	
			White percent 1976	.38***	White percent 1976	.26**	
1980 Conservative-Liberal	4	2.74**	Handedness	.34**	Handedness	.17 (*p* = .084)	.99
			Conscientiousness	−.60***	Conscientiousness	−.41***	
			Agreeableness 1980	.41**	Agreeableness 1980	.31**	
			Income 1980	.30**	Income 1980	.22*	
1984 Conservative-Liberal	4	1.69*	Handedness	.56***	Handedness	.42***	3.00
1964 Democratic-Republican	6	2.42**	Handedness	.40***	Handedness	.35***	2.45
			White percent 1964	.62**	White percent 1964	.56***	
			Urbanization 1964	.17*	Urbanization 1964	.16*	
1972 Democratic-Republican	6	2.90**	Handedness	.38***	Handedness	.29**	.08
			White percent 1972	.48***	White percent 1972	.30**	
			Urbanization 1972	.23*	Urbanization 1972	.21*	
1976 Democratic-Republican	6	2.11*	Neuroticism	.35*	Neuroticism	.27*	.23
			White percent 1976	−.41**	White percent 1976	−.28*	
1980 Democratic-Republican	6	1.82*	Neuroticism	.47***	Neuroticism	.38**	.67
			White percent 1976	−.30**	White percent 1976	−.24*	
1984 Democratic-Republican	6	2.26*	Handedness	.34*	Handedness	.30*	1.39
			Neuroticism	.31*	Neuroticism	.23 (*p* = .088)	
1988 Democratic-Republican	6	2.76**	Handedness	.39**	Handedness	.30*	.46
1964 Democratic-Republican	7	2.32*	Handedness	.27**	Handedness	.26**	3.52
			White percent 1964	.45***	White percent 1964	.44***	
			Ideology 1964	.33**	Ideology 1964	.32**	
1976 Democratic-Republican	7	2.11*	Neuroticism	.35*	Neuroticism	.27*	.23
			White percent 1976	−.41**	White percent 1976	−.28*	
1980 Democratic-Republican	7	2.33**	Neuroticism	.38***	Neuroticism	.30**	.00
			White percent 1980	−.48***	White percent 1980	−.41***	
			Ideology 1980	.44**	Ideology 1980	.42***	
1984 Democratic-Republican	7	2.59**	Neuroticism	.25*	Neuroticism	.19 (*p* = .097)	3.23
			Ideology 1984	.56***	Ideology 1984	.54***	
							
2000 Democratic-Republican	7	2.05*	Handedness	.48***	Handedness	.38***	.49
			Urbanization 2000	.21**	Urbanization 2000	.20**	
			Ideology 2000	.41***	Ideology 2000	.37***	
2008 Democratic-Republican	7	1.66*	Handedness	.22*	Handedness	.22*	1.48
			Urbanization 2008	.26***	Urbanization 2008	.26**	
			Ideology 2008	.66***	Ideology 2008	.65***	
2016 Democratic-Republican	7	2.29*	Openness	.15*	Openness	.14*	4.41*
			Urbanization 2016	.26***	Urbanization 2016	.27***	
			Ideology 2016	.70***	Ideology 2016	.72***	

^a^Moran’s I statistic standard deviate.

^b^LM (Lagrange Multiplier) test for residual autocorrelation.

*p < .05. **p < .01. ***p < .001. All tests are two-tailed.

### Supplementary Simultaneous Multiple Regression Analyses

Although the logically chosen multiple regression strategy which included the stepwise selection of control variables is thought to be most appropriate for the present research context, corresponding supplementary multiple regression equations also were computed for comparison purposes that used unrestricted simultaneous entry of control variables. Supplementary Table 5a displays the results corresponding to Table 5 with political ideology as the criterion. Regarding handedness, equations in Supplementary Table 5a show that handedness was dropped as an independent predictor for the elections of 1980, 1984, 1992, and 1996, and was reduced to a significance level of .051 for 1964. Equations in Supplementary Table 7a, which correspond to those in Table 7 for Democratic-Republican popular vote as the criterion, show that handedness was dropped as an independent predictor for the elections of 1980, 1984, and 1988, and was reduced to a significance level of .058 for 1992. Equations in Supplementary Table 8a, which correspond to those in Table 8, show no changes in the status of handedness as a predictor for any of the elections from 1964 to 2016.

## Discussion

Individually analyzing each of the U.S. presidential elections of the past 60 years using separate Conservative-Liberal and Democratic-Republican outcome variables in the present study yielded essentially the same intriguing pattern of results found by McCann (2018a) who employed a composite ideology-party preference measure based on the 1996 to 2012 contests. Higher state levels of left-handedness were associated significantly with liberal ideology in each of the presidential election years from 1964 to 2016. Comparable ideology data were not available for 2020 (see Table 4). Higher state levels of left-handedness also were associated with a greater degree of Democratic candidate support in each of the presidential election years from 1964 to 2020 except for 1976 (see Table 6). The magnitude of the relations between state-level handedness and the two political preference variables was not trivial. The mean size of the 28 significant Pearson correlations involving the two political criteria was .62 (*SD* = .12) with a range of .38–.80. This indicates that handedness alone could account for a mean of 40.1% (*SD* = 14.9) of the variance in the two political preference variables.

Furthermore, when the Big Five, White population percent, urbanization, and income variables were given the opportunity to enter the corresponding multiple regression equations along with handedness when the Pearson correlation between handedness and either of the two political criteria was significant, handedness still emerged with a significant regression coefficient in 26 of the 28 equations (see Table 9). The two exceptions occurred for 1968 with either ideology or party choice as the outcome variable. In contrast, one or more Big Five personality variables only produced a significant regression coefficient in nine of the 28 equations, White population percent in nine, urbanization in nine, and income in six. In fact, one or more of these other eight potential independent predictors only surfaced as such in 21 of the 28 equations compared to all 28 for handedness. As well, although handedness was a potent predictor of both Conservative-Liberal ideology and Democratic-Republican presidential popular vote, and ideology was an independent predictor of party preference in 12 of the 14 election years tested, handedness still retained its independent capacity to predict party preference even when ideology was statistically controlled in seven election years (see Table 9). The robust capacity of handedness as a predictor in this context is evident.

One notable difference between the present results and the work of McCann (2018a) concerns the capacity of handedness as a statistical control to eliminate significant prediction of the two political orientation outcome variables by Big Five personality variables. McCann (2018a) found that including handedness in the multiple regression equation eliminated the Big Five as significant predictors using the 1996–2012 the composite criterion of political preference. However, the present study shows that for the 1968, 1976, 1980, and 1988 elections, conscientiousness remained a significant predictor of the ideology criterion, and, for 1980 and 1988, agreeableness remained so (see Table 9). Neuroticism also survived as an independent predictor of Democratic-Republican popular vote for 1968, 1976, 1980, 1984, 1992, 1996 without ideology considered as a predictor. In fact, neuroticism still survived as a significant independent predictor in 1968, 1976, 1980, 1984, and 1996 with ideology treated as a covariate. As well, openness also emerged as an independent contributor in 2020 with ideology controlled.

Therefore, even though the correlations between handedness and the Big Five are invariable over the two studies, perhaps certain conditions pertained that allowed one or more of these four Big Five personality variables to surface as an independent predictor of political orientation in 1968, 1976, 1980, 1984, 1988, 1992, 1996, and 2020. Note that except for the emergence of neuroticism as an independent predictor of the Democratic-Republican criterion in 1996 and likewise the emergence of openness in 2020, the other six years—1968, 1976, 1980, 1984, 1988, and 1992—were not included in the McCann (2018a) composite criterion. Also note that here the potent nature of handedness as an independent predictor of ideology in the 1996–2016 period is underlined by the fact that 29 of the 30 partial correlations attained significance, the exception occurring for 1996. As well, all 35 partial correlations for the 1996–2020 period were significant with the Democratic-Republican vote criterion (see Table 6). In contrast, prior to 1996, 32 of the 40 partial correlations were significant with ideology as the criterion and 25 of the 40 were significant with the Democratic-Republican popular vote criterion.

Generally, the Pearson and partial correlation results show stronger relations of handedness to the two political orientation variables during more recent years. In fact, from 1984 onward, handedness has the largest regression coefficient with either ideology or presidential candidate preference as the criterion. These results suggest that whatever underlying political differences are fundamentally related to overt and latent left-handedness have become more apparent with increasing political polarization and the greater alignment of ideology and political partisanship (e.g., Abramowitz & Saunders, 1998; Wright & Birkhead, 2012).

According to the explanatory suggestions put forth by McCann (2018a), such relations are rooted in hypothesized but poorly understood genetic links between handedness, personality, and political beliefs and attitudes. However, the original speculations were qualified by a strong cautionary stance emphasizing that researchers are unlikely to find direct evidence that handedness predicts political preferences in conventional studies in which individuals are the units of analysis. This was deemed probable because “latent or hidden relations may exist between handedness, political orientation, and personality that depend upon both the direct and more tangential genetics of handedness” (McCann, 2018a, p. 308). It was speculated that other genetic or nongenetic influences might produce lower levels of *overt* left-handedness in a population than would be expected from the postulated underlying genetic network. The impacts of the foundational *genetic predisposition* to left-handedness in a population on the correlates of handedness may be much greater than the impacts that can be attributed to the *overt* levels of left-handedness in that population. McCann (2018a) refers to this possibility as the “iceberg effect” (p. 308).

McCann (2018a) also speculated that the five pathways articulated by Rentfrow et al. (2008) to show how aggregate-level phenomena might spring from individual-level processes can be used to explain how state-level relations involving overt or latent left-handedness may be enhanced even when there is only a modest relation to political preferences and personality. Consider the following examples. Pathway 1: If the ratio of right-handed to left-handed persons is relatively higher in a state, then there likely will be correspondingly greater indications of right-handed psychological and behavioral manifestations in that state such as lower neuroticism, lower openness, and a more conservative and Republican electorate. Pathway 2: If a state has such prominent right-handed psychological and behavioral manifestations, then conservative social structures and institutions eventually will develop that cater to those manifestations. Pathway 3: The psychosocial climate in such a state is likely to promote conformity to those characteristics even among those who are higher on neuroticism and openness, pushing them to *act* in a less neurotic, less open, and more conservative manner. Pathway 4: Conservative state institutional and social structures also can enhance the opportunities for conservatives, right-handers, the less neurotic, and the less open, and limit the opportunities for those with elements of the opposite profile. Pathway 5: The social norms of a conservative state can influence individual difference levels through socialization processes by fostering conservatism, lower neuroticism, and lower openness, and, through residential mobility by retaining and attracting conservatives, the less neurotic, and the less open, while losing and repelling liberals, the more neurotic, and the more open.

McCann (2018a) speculated too on potential reasons for relations between handedness, personality, and political leaning at the individual level of analysis. The focus was on studies suggesting that left-handers have the capacity for better communication between brain hemispheres (e.g., Cherbuin & Brinkman, 2006; Hayashi et al., 2008; Wiberg et al., 2019) because of genetically based corpus callosum differences (e.g., Gorynia & Egenter, 2000; Habib et al., 1991; Ocklenburg et al., 2014) in size and neurological transmission capacity (e.g., Luders et al., 2010; Propper et al., 2012; Westerhausen et al., 2004). These corpus callosum differences leading to poorer interhemispheric communication are related to conservative characteristics such as greater lack of tolerance and higher cognitive inflexibility (e.g., Cherbuin & Brinkman, 2006; Jasper et al., 2014; Sontam et al., 2009). McCann (2018a) also suggested that superior interhemispheric interaction and the accompanying corpus callosum structural characteristics might be more pronounced if research samples also included participants who are latently rather than overtly predisposed to left-handedness, perhaps as suggested by their political orientation and their standing on the Big Five personality dimensions.

Caution should be exercised regarding any cross-level interpretive extrapolations (e.g., Rentfrow et al., 2008). One commits the “ecological fallacy” when it is *assumed* that aggregate-level relations also pertain to the individual level (Robinson, 1950), and conversely, one commits the “compositional fallacy” when it is *assumed* that individual-level relations also pertain to the aggregate level (Pettigrew, 1997). Similar relations might not be found at each level of analysis. Without empirical evidence of a relation at both levels, it can only be *assumed* that a relation is common to both levels, and that assumption is open to the possibility of interpretational error. Aggregate-level and individual-level relations may be logically independent.

Furthermore, even if there is demonstrated empirical consistency in the relations across levels, the factors contributing to that parallel nature still might be logically independent. Unfortunately, there exists a paucity of individual-level research to offer any corroboration of the present state-level results. The interpretational dilemma in the present context is additionally complicated because of the speculation that it is not necessarily only *overt* handedness that is involved but also the concept of *latent* handedness. Consequently, due caution in interpretation is advised regarding the potential generalization of the results reported here from the state to the individual level of analysis.

The supplementary multiple regression analyses were conducted to determine whether simultaneous entry of the Big Five followed by the simultaneous entry of income, White population percent, and urban population percent would produce *substantively* different results than the main analyses which used the stepwise mode to select variables from each of the two blocks of potentially significant predictors. However, the Supplementary Table 5a and Table 7a results using simultaneous rather than stepwise regression still demonstrated substantial support for the general relation between handedness and political leaning. Using simultaneous regression reduced the number of election years that handedness was a significant predictor of ideology from 13 of 14 to nine of 14, and to eight of 14 if the *p* value of .051 for 1964 is strictly viewed as nonsignificant. Similarly, using simultaneous regression reduced the number of election years that handedness was a significant predictor of Democratic-Republican preference from 12 of 15 to nine of 15, and to eight of 15 if the *p* value of .058 for 1992 is strictly viewed as nonsignificant. But, of course, the simultaneous regression solutions included variables as predictors that were not statistically significant, thereby reducing relations between handedness and political preference by cumulatively removing variance from the two political preference criteria and reducing the degrees of freedom for the significance tests. To the author, the original multiple regression models using stepwise selection remain the most suitable for the purposes of the present research.

### Limitations and Issues

The use of the sole assessment of state levels of left-handedness based on data collected through a procedure that may not have been based on samples that were particularly representative of state populations might be perceived as a weakness of the present study (McCann, 2018a). So might the fact that the initial data were from 1986, although state inbound and outbound residential mobility are not likely to have had appreciable or meaningful effects on the underlying state gene pools thought to be responsible for state differences in handedness. Nonetheless, despite these apparent shortcomings, relations between this state handedness variable and political preference variables were strong and consistent over the years from 1964 to 2020. In fact, they were even somewhat more so during the past two decades. Therefore, if anything, the handedness measure seems to warrant bolstered confidence.

Readers might also be concerned that the state Big Five personality scores are based on the years from 1999 to 2005 while other variables in the present study pertain to the years 1964–2020. However, the temporal stability and relations to several other sociodemographic variables at least from 1999 to 2015 has been shown by Elleman et al. (2018), the years of analysis in their research. Furthermore, the validity of the state Big Five scores has been demonstrated by their successful use in a wide variety of research contexts (e.g., Ebert et al., 2022; McCann, 2021a, 2021, 2022a, 2022b, 2023; Obschonka et al., 2013; Rentfrow et al., 2008, 2009). The state scores also have proven useful in published studies focused on years before 1999 (e.g., McCann, 2019) and after 2015 (e.g., McCann, 2022a).

Some readers also might have concerns that although variable assessment generally was strong, the samples on which variables were based lacked congruency. That is, the variables tended to be generated from published academic research and official government data, but the same persons were not necessarily or often in the different state samples on which the data for the variables were based. However, such a lack of congruency is likely to work against finding significant relations between the state variables. Again, confidence in the effectiveness of the assessments is enhanced by the fact that many expected significant relations indeed were found in the present study despite this lack of congruence.

The effective sample size of 48 can be judged as relatively small by the standards usually applied in conventional research when the sample is a representative subset of a population. However, the population of interest in the current work is the 48 contiguous states, and so is the sample. Therefore, although a sample size of 48 in a more conventional study would justifiably raise warning signals, for example, that the resulting β regression coefficients might be unstable, such potential inferential errors are not readily applicable in the present context. Comparable analyses have been reported for many similarly sized samples in the past (e.g., Barber, 2015; Erikson et al., 1993; McCann, 1992, 1997, 2008; Pesta & McDaniel, 2014). Furthermore, the necessity of any “inferential” statistics in this circumstance is questionable (e.g., see Simonton, 1984, Appendix A).

As noted in the current introductory section, analyses of variables based on aggregated units often yield larger correlations and regression coefficients than analyses based on variables with individuals as the units of analysis (e.g., Erikson et al., 1993; Ostroff, 1993). Aggregation tends to cancel out the degree of measurement error for each aggregated unit. As Jackson et al. (2003) have stated, “aggregation as a general methodology is not necessarily a strange thing to do, when it is remembered that individual level data are themselves based on aggregated item level data” (p. 399). The process does cancel out measurement errors and improve reliability. As Ostroff (1993) pointed out, measurement error attenuates individual-level correlations and thereby increases the ratio of aggregate-level correlations to individual-level correlations to the degree that it exists. Furthermore, to the extent that it is assumed that the same processes and relations are operating at both the aggregate and the individual level, and other factors such as individual-level measurement error are accounted for, then the correlations at the two levels should be similar. Although the effect sizes reported here are valid for states in the current study, readers should be aware of this procedural impact on the magnitude of Pearson correlations and b coefficients when interpreting the results of the present study.

Of course, this is a cross-sectional correlational study for each of the 15 presidential elections from 1964 to 2020, and as such, no causal inferences can be drawn from the results. For example, handedness correlates with political preferences and predates the development of political beliefs and attitudes in the trajectory of life. However, there is no empirical evidence that can be gleaned from the present study to support *any* causal inferences even though *speculations* may suggest such causal links.

It also should be reiterated here that the *speculation* of an underlying genetic framework with some common components related to handedness, personality, and political preference has not received sufficient empirical research attention to date to provide much in the way of confirmation or disconfirmation. There is research indicating some genetic commonalities between Big Five personality and political preference (e.g., Eaves et al., 1989; Kandler et al., 2015; Verhulst et al., 2012) and between neuroticism and handedness (Cuellar-Partida et al., 2021). However, genetic commonalities between political preference and handedness, or between personality, political preference, and handedness, apparently have not been the subject of study. Nor has the *speculation* that “latent or hidden relations may exist between handedness, political orientation, and personality that depend upon both the direct and more tangential genetics of handedness” (McCann, 2018a, p. 308) been researched. Therefore, these tentative explanatory mechanisms suggested by McCann (2018a) must largely remain in the realm of speculation pending further research.

### Potential Ramifications for Application

Although the present research and its direct predecessor (i.e., McCann, 2018a) have focused on political preference—an area of potential opportunity for application, both studies were initiated and conducted from the basic or pure science perspective rather than as applied science research projects. Consequently, it is premature to offer applied recommendations. However, it does seem appropriate to suggest potential limited ramifications of the present research results for applications in this political context.

For example, because the state gene pools that largely produce the state level of left-handedness have high inherent temporal stability, a relatively impervious barrier to wholesale ideological change may exist that is rather resistant to the persuasive appeals of politicians and political pundits. Of course, these same factors also make it easier for those with persuasive appeals that are in line with the predominant existing political views in a state to maintain their constituencies. Perhaps it might be advantageous for political scientists, politicians, and political pundits to be aware of these underlying factors, to know the extent in any constituency to which such factors may make it more or less likely that they will gain support, and to tailor their messages and campaign efforts accordingly. However, the full value of this line of inquiry will only become apparent with much additional empirical study.

### Future Research

Further research at both the state and the individual level obviously is necessary to more fruitfully address the underlying dynamics that might explain the surprisingly potent predictive capacity of state levels of left-handedness regarding political ideology and political party preference. At the state level, a more exhaustive search for variables that could contribute to an explanation of the relation between handedness and political persuasion might prove beneficial. As it stands, it is quite evident that the statistical control of state Big Five personality variables, income, White population percent, and urban population percent does not negate the relation between handedness and political choices. What other state-level variables might have the requisite explanatory power is unknown at this time.

At the individual level, a more concerted effort to examine the potential association between left-handedness and contemporary political liberalism in appropriate samples would be useful in perhaps eventually providing keys to the explanation of handedness-politics relations both at the individual and the state level of analysis. Of course, such research could be conducted in several diverse realms such as the quest for genetic links and the relations of handedness to the adoption of elemental political beliefs and attitudes in individual and cultural worldviews. Of special benefit would be studies directed at the speculated existence of *latent* handedness and its correlates.

It has been suggested (e.g., Rentfrow, 2010) that multilevel modeling procedures (e.g., Hox & Roberts, 2010) could be employed to determine whether relational patterns between individual difference variables and outcome indicators vary or are constant across different levels of analysis. However, the complexity and cost of such an approach with individual and state relations within the current research context would be prohibitively steep for most research programs. Such a multiple regression strategy would require that *all* key variables for a particular study are first measured at the individual level for *each* participant in large representative samples from each state, a rather daunting order. Scores must be available on every variable for each person. Scores collected from other respondents cannot be substituted. Nevertheless, perhaps future researchers can overcome the obstacles and eventually conduct an appropriate study in this vein to enhance the knowledge base regarding the relations between personality, political orientation, and handedness.

### Conclusion

The present research has shown that higher state-level estimates of left-handedness have been robustly associated with liberal ideology and Democratic political preferences in the presidential election years from 1964 to 2020. Although speculations have been made regarding why this association occurs, an answer ultimately remains a mystery. Further research with both individuals and states is needed to bolster the validity of the speculative assertions that have been offered, or to offer other avenues of explanation and interpretation for this inherently interesting phenomenon. It seems fundamentally important in a nation founded on the principles of democracy to know whether political ideology and electoral choice are at least partially grounded in genetic predispositions manifested through handedness, the extent of that impact, and why it occurs.

## Supplemental Material

Supplemental Material - State Resident Handedness, Ideology, and Political Party Preference: U.S. Presidential Election Outcomes Over the Past 60 Years Supplemental Material for State Resident Handedness, Ideology, and Political Party Preference: U.S. Presidential Election Outcomes Over the Past 60 Years by Stewart J. H. McCann in Psychological Reports.

## Data Availability

The data are available at https://osf.io/k3drq/?view_only=42eb181e7f2546d2b691b17a6a433b1a
