# Supplementary material for: State Resident Handedness, Ideology, and Political Party Preference: U.S. Presidential Election Outcomes Over the Past 60 Years
Source: Psychol Rep. 2024 Jan 12;129(1):327–71. doi: 10.1177/00332941241227521 (PMC12717291; doi:10.1177/00332941241227521)
Supplement: Supplemental Material - State Resident Handedness, Ideology, and Political Party Preference: U.S. Presidential Election Outcomes Over the Past 60 Years [file sj-pdf-1-prx-10.1177_00332941241227521.pdf]

Supplementary Table 5a

Simultaneous Multiple Regression Equations Demonstrating the Relation of Handedness, Big Five Personality, Income, White Population Percent, and Urbanization to Conservative-Liberal Ideology 1964-2016

Year	Step	Entry	Predictor pool	df	R^2 change	*F*	Significant predictors	β	*t*
1964	1	forced	Handedness	1, 46	.292	18.96***	White percent 1964	.59	5.26***
	2	forced	Big Five	5, 41	.109	1.49	Handedness	.27	2.02 (*p* = .051)
	3	forced	Pool of three[a]	3, 38	.292	12.01***	Neuroticism	.24	2.01 (*p* = .051)
1968	1	forced	Handedness	1, 46	.278	17.68***	White percent 1968	.56	5.98***
	2	forced	Big Five	5, 41	.142	2.01	Income 1968	.33	2.11*
	3	forced	Pool of three	3, 38	.359	20.55***	Neuroticism	.32	3.08**
							Agreeableness	.25	2.26*
							Conscientiousness	-.22	-2.23*
1972	1	forced	Handedness	1, 46	.329	22.60***	White percent 1972	.58	5.83***
	2	forced	Big Five	5, 41	.115	1.70	Handedness	.32	2.62*
	3	forced	Pool of three	3, 38	.299	14.74***			
1976	1	forced	Handedness	1, 46	.388	29.19***	White percent 1976	.43	4.43***

	2	forced	Big Five	5, 41	.187	3.62**	Conscientiousness	-.33	-2.56*
	3	forced	Pool of three	3, 38	.185	9.82***	Agreeableness	.29	2.47*
							Neuroticism	.27	2.46*
							Handedness	.24	2.16*
1980	1	forced	Handedness	1, 46	.315	21.14***	Conscientiousness	-.48	-3.11**
	2	forced	Big Five	5, 41	.247	4.63**	Agreeableness	.38	2.77**
	3	forced	Pool of three	3, 38	.107	4.12*	White percent 1980	.29	2.45*
1984	1	forced	Handedness	1, 46	.314	21.09***	(none)		
	2	forced	Big Five	5, 41	.126	1.85			
	3	forced	Pool of three	3, 38	.068	1.74			
1988	1	forced	Handedness	1, 46	.384	28.67***	Conscientiousness	-.44	-2.65*
	2	forced	Big Five	5, 41	.194	3.78**	Agreeableness	.32	2.16*
	3	forced	Pool of three	3, 38	.023	.75	Handedness	.32	1.88 (*p* = .068)
1992	1	forced	Handedness	1, 46	.333	22.96***	White percent 1992	.31	2.36*
	2	forced	Big Five	5, 41	.138	2.14			
	3	forced	Pool of three	3, 38	.111	3.36*			

1996	1	forced	Handedness	1, 46	.332	22.90***	Extraversion	.41	2.32*
	2	forced	Big Five	5, 41	.152	2.41	Openness to Experience	.40	2.13*
	3	forced	Pool of three	3, 38	.048	1.30			
2000	1	forced	Handedness	1, 46	.405	31.37***	Extraversion	.40	2.42*
	2	forced	Big Five	5, 41	.150	2.78*	Conscientiousness	-.39	-2.20*
	3	forced	Pool of three	3, 38	.015	.44	Handedness	.38	2.29*
2004	1	forced	Handedness	1, 46	.505	46.86***	Handedness	.46	2.87**
	2	forced	Big Five	5, 41	.109	2.32	Extraversion	.33	2.16*
	3	forced	Pool of three	3, 38	.011	.36			
2008	1	forced	Handedness	1, 46	.587	65.50***	Handedness	.59	4.45***
	2	forced	Big Five	5, 41	.075	1.81	Extraversion	.30	2.05*
	3	forced	Pool of three	3, 38	.008	.31			
2012	1	forced	Handedness	1, 46	.543	54.67***	Handedness	.56	3.96***
	2	forced	Big Five	5, 41	.051	1.04			
	3	forced	Pool of three	3, 38	.022	.74			

2016	1	forced	Handedness	1, 46	.644	83.33***	Handedness	.59	5.15***
	2	forced	Big Five	5, 41	.040	1.03			
	3	forced	Pool of three	3, 38	.068	3.47*			

[a]The pool of three potential predictors included the year-appropriate income, urbanization, and White percent variables.

*$p < .05$. **$p < .01$. ***$p < .001$. All tests are two-tailed.

Supplementary Table 7a

Simultaneous Multiple Regression Equations Demonstrating the Relation of Handedness, Big Five Personality, Income, White Population Percent, and Urbanization to Democratic-Republican Presidential Vote 1964-2020

Year	Step	Entry	Predictor pool	df	R^2 change	*F*	Significant predictors	β	*t*
1964	1	forced	Handedness	1, 46	.323	21.99***	White percent 1964	.70	7.26***
	2	forced	Big Five	5, 41	.065	.87	Handedness	.27	2.33*
	3	forced	Pool of three[a]	3, 38	.385	21.43***	Neuroticism	.23	2.20*
1968	1	forced	Handedness	1, 46	.215	12.58***	Conscientiousness	-.54	-3.28**
	2	forced	Big Five	5, 41	.283	4.61**	Urban percent 1968	.44	2.24*
	3	forced	Pool of three	3, 38	.078	2.32	Neuroticism	.32	2.23*
1972	1	forced	Handedness	1, 46	.261	16.21***	White percent 1972	.42	3.29**
	2	forced	Big Five	5, 41	.137	1.86	Handedness	.30	1.84 (*p* = .073)
	3	forced	Pool of three	3, 38	.170	4.98**			
1976	1	forced	Handedness	1, 46	.031	1.49	White percent 1976	-.44	-2.98**
	2	forced	Big Five	5, 41	.260	3.00*			
	3	forced	Pool of three	3, 38	.175	4.14*			

1980	1	forced	Handedness	1, 46	.144	7.73**	Neuroticism	.40	2.77**
	2	forced	Big Five	5, 41	.320	4.89***	White percent 1980	-.40	-2.96**
	3	forced	Pool of three	3, 38	.104	3.03*			
1984	1	forced	Handedness	1, 46	.215	12.57 ***	(none)		
	2	forced	Big Five	5, 41	.169	2.26			
	3	forced	Pool of three	3, 38	.014	.29			
1988	1	forced	Handedness	1, 46	.156	8.48**	(none)		
	2	forced	Big Five	5, 41	.087	.94			
	3	forced	Pool of three	3, 38	.040	.71			
1992	1	forced	Handedness	1, 46	.376	27.66***	Handedness	.36	1.95 (*p* = .058)
	2	forced	Big Five	5, 41	.101	1.58			
	3	forced	Pool of three	3, 38	.002	.06			
1996	1	forced	Handedness	1, 46	.548	55.86***	Handedness	.50	3.64***
	2	forced	Big Five	5, 41	.148	3.98**	Conscientiousness	-.38	-2.69*
	3	forced	Pool of three	3, 38	.016	.73	Neuroticism	.28	2.44*

2000	1	forced	Handedness	1, 46	.598	68.43***	Handedness	.51	4.37***
	2	forced	Big Five	5, 41	.089	2.32	Neuroticism	.25	2.58*
	3	forced	Pool of three	3, 38	.097	5.71**			
2004	1	forced	Handedness	1, 46	.629	78.11***	Handedness	.51	3.79***
	2	forced	Big Five	5, 41	.066	1.77			
	3	forced	Pool of three	3, 38	.043	2.10			
2008	1	forced	Handedness	1, 46	.588	65.66***	Handedness	.67	5.02***
	2	forced	Big Five	5, 41	.044	.98			
	3	forced	Pool of three	3, 38	.036	1.39			
2012	1	forced	Handedness	1, 46	.584	64.53***	Handedness	.63	5.06***
	2	forced	Big Five	5, 41	.039	.85			
	3	forced	Pool of three	3, 38	.076	3.22*			
2016	1	forced	Handedness	1, 46	.476	41.78***	Handedness	.49	4.56***
	2	forced	Big Five	5, 41	.159	3.58**	White percent 2016	-.26	-2.32*
	3	forced	Pool of three	3, 38	.150	8.80***	Agreeableness	.22	2.04*

2020	1	forced	Handedness	1, 46	.489	44.04***	Handedness	.51	4.52***
	2	forced	Big Five	5, 41	.136	2.99*	Income 2020	.25	2.08*
	3	Forced	Pool of three	3, 38	.136	7.20***			

[a]The pool of three potential predictors included the year-appropriate income, urbanization, and White percent variables.

*p < .05. **p < .01. ***p < .001. All tests are two-tailed.

Supplementary Table 8a

Simultaneous Multiple Regression Equations Demonstrating the Relation of Conservative-Liberal Ideology to Democratic-Republican Presidential Vote 1964-2020 When Appended to the Predictors with Significant βs in the Equations of Table 7

Year	Step	Entry	Predictor pool	df	R^2 change	*F*	Significant predictors	β	*t*
1964	1	forced	Handedness	3, 44	.737	41.17***	White percent 1964	.45	4.82***
			White percent 1964				Ideology 1964	.33	2.92**
			Urbanization 1964				Handedness	.27	3.10**
	2	forced	Ideology 1964	1, 43	.044	8.55**			
1968	1	forced	Handedness	3, 44	.430	11.05***	Neuroticism	.48	3.77***
			Neuroticism						
			Urbanization 1968						
	2	forced	Ideology 1968	1, 43	.012	.96			
1972	1	forced	Handedness	3, 44	.530	16.51***	Ideology 1972	.75	5.26***
			White percent 1972						
			Urbanization 1972						
	2	forced	Ideology 1972	1, 43	.184	27.64***			

1976	1	forced	Handedness	3, 44	.373	8.74***	White percent 1976	-.47	-2.91**
			Neuroticism				Neuroticism	.32	2.26*
			White percent 1976						
	3	forced	Ideology 1976						
1980	1	forced	Handedness	3, 44	.489	14.04**	White percent 1980	-.48	-4.16***
			Neuroticism				Ideology 1980	.44	3.30**
			White percent 1980				Neuroticism	.38	3.43***
	2	forced	Ideology 1980	1, 43	.103	10.87**			
1984	1	forced	Handedness	2, 45	.296	9.46***	Ideology 1984	.56	4.32***
			Neuroticism				Neuroticism	.25	2.12*
	2	forced	Ideology 1984	1, 43	.210	18.69***			
1988	1	forced	Handedness	1, 46	.154	8.34**	Ideology 1988	.83	6.70***
	2	forced	Ideology 1988	1, 45	.422	44.83***			
1992	1	forced	Handedness	2, 45	.428	16.84***	Ideology 1992	.38	2.94**
			Neuroticism				Handedness	.31	2.38*
	2	forced	Ideology 1992	1, 44	.094	8.65**			

1996	1	forced	Handedness	2, 45	.619	36.57***	Ideology 1996	.47	5.20***
			Neuroticism				Handedness	.37	3.99***
	2	forced	Ideology 1996	1, 44	.145	27.02***	Neuroticism	.25	3.18**
2000	1	forced	Handedness	2, 45	.671	45.97***	Handedness	.48	5.03***
			Urbanization 2000				Ideology 2000	.41	4.23***
	2	forced	Ideology 2000	1, 44	.095	17.89***	Urbanization 2000	.21	2.70**
2004	1	forced	Handedness	2, 45	.667	45.13***	Ideology 2004	.59	6.38***
			Income 2004				Handedness	.31	3.21**
	2	forced	Ideology 2004	1, 44	.160	40.76***			
2008	1	forced	Handedness	2, 45	.638	39.57***	Ideology 2008	.66	6.54***
			Urbanization 2008				Urbanization 2008	.26	3.98***
	2	forced	Ideology 2008	1, 44	.179	42.70***	Handedness	.22	2.17*
2012	1	forced	Handedness	2, 45	.640	39.98***	Ideology 2012	.66	7.37***
			Urbanization 2012				Urbanization 2012	.24	3.94***
	2	forced	Ideology 2012	1, 44	.199	54.36***	Handedness	.23	2.53*

2016	1	forced	Handedness	3, 44	.714	36.62***	Ideology 2016	.70	6.73***
			Openness				Urbanization 2016	.26	3.72***
			Urbanization 2016				Openness to Experience	.15	2.02*
	2	forced	Ideology 2016	1, 43	.147	45.32***			

*p < .05. **p < .01. ***p < .001. All tests are two-tailed.
